# G9a is essential for EMT-mediated metastasis and maintenance of cancer stem cell-like characters in head and neck squamous cell carcinoma

**DOI:** 10.18632/oncotarget.3159

**Published:** 2015-01-29

**Authors:** Shuli Liu, Dongxia Ye, Wenzheng Guo, Wenwen Yu, Yue He, Jingzhou Hu, Yanan Wang, Ling Zhang, Yueling Liao, Hongyong Song, Shuangshuang Zhong, Dongliang Xu, Huijing Yin, Beibei Sun, Xiaofei Wang, Jingyi Liu, Yadi Wu, Binhua P. Zhou, Zhiyuan Zhang, Jiong Deng

**Affiliations:** ^1^ Department of Oral and Maxillofacial–Head and Neck Oncology, Ninth People's Hospital, Shanghai Jiao Tong University School of Medicine, Shanghai, China; ^2^ Key Laboratory of Cell Differentiation and Apoptosis of Chinese Minister of Education, Shanghai Jiao Tong University School of Medicine, Shanghai, China; ^3^ Shanghai Key Laboratory for Tumor Microenvironment and Inflammation, Shanghai Jiao Tong University School of Medicine, Shanghai, China; ^4^ Translation Medicine Center, Shanghai Chest Hospital, Shanghai Jiao Tong University, Shanghai, China; ^5^ Department of Molecular and Cellular Biochemistry, Markey Cancer Center, University of Kentucky College of Medicine, Lexington, KY, USA

**Keywords:** HNSCC, EMT, lymph node metastasis, cancer stem cell, G9a

## Abstract

Head and neck squamous cell carcinoma (HNSCC) is a particularly aggressive cancer with poor prognosis, largely due to lymph node metastasis and local recurrence. Emerging evidence suggests that epithelial-to-mesenchymal transition (EMT) is important for cancer metastasis, and correlated with increased cancer stem cells (CSCs) characteristics. However, the mechanisms underlying metastasis to lymph nodes in HNSCC is poorly defined. In this study, we show that E-cadherin repression correlates with cancer metastasis and poor prognosis in HNSCC. We found that G9a, a histone methyltransferase, interacts with Snail and mediates Snail-induced transcriptional repression of E-cadherin and EMT, through methylation of histone H3 lysine-9 (H3K9). Moreover, G9a is required for both lymph node-related metastasis and TGF-β-induced EMT in HNSCC cells since knockdown of G9a reversed EMT, inhibited cell migration and tumorsphere formation, and suppressed the expression of CSC markers. Our study demonstrates that the G9a protein is essential for the induction of EMT and CSC-like properties in HNSCC. Thus, targeting the G9a-Snail axis may represent a novel strategy for treatment of metastatic HNSCC.

## INTRODUCTION

Head and neck squamous cell carcinoma (HNSCC) is the sixth most common cancer in the world with an annual incidence of over 560,000 cases [1]. Despite a variety of advances in combined-modality treatments, survival for HNSCC patients has been remained poor over the past two decades, due in large part to uncontrolled metastasis and local recurrence. HNSCC metastasis is primarily to lymph nodes [2] and the most important adverse prognostic factor in HNSCC is metastasis to cervical lymph nodes [3–5]. Certain genetic expression profiles in the primary tumor might predict the incidence of lymph node metastasis [6]. This finding suggests that genetic factors might direct early metastatic cells to lymph nodes in head and neck. Thus, identification of the genetic and molecular factors involved in this metastatic process are crucial to advance our understanding of HNSCC metastasis, and for insight into the development of therapeutic strategies to improve survival in HNSCC patients.

Emerging evidence suggests that epithelial-mesenchymal transition (EMT) plays an important role in cancer metastasis. EMT is an essential phenotypic conversion during embryonic development, tissue remodeling, wound healing, and cancer metastasis [7–9]. During EMT, cells loose epithelial characteristics such as cell polarity and cell-cell contact, and gain mesenchymal properties such as motility [10]. EMT is a dynamic and reversible process, and is provoked by signals from the microenvironment [11–13] such as TGF-β, Wnt, and TNFα [14, 15]. TGF-β, a pluripotent factor, [16] is a significant element for EMT induction in epithelial cells during embryonic development and cancer progression [7]. This factor stimulates cancer cells to become both motile and invasive, thereby leaving the primary tumor site, and disseminate to distant sites of the body. Importantly, when metastatic cancer cells migrate, the signals encountered are different from those obtained in the primary tumor, and cells can revert to an epithelial state by mesenchymal-epithelial transition (MET) [8, 17, 18].

A hallmarker of EMT is lost of E-cadherin expression [8]. In fact, decreased or complete loss of E-cadherin expression is associated with metastasis to lymph nodes and poor prognosis in HNSCC [19]. Promoter methylation, mediated by E-box-binding transcription repressors such as Snail, Twist and Slug, likely impacts on the repression of E-cadherin during EMT [20]. In fact, upregulated Snail correlates with local recurrence in HNSCC [21]. Snail can induce epigenetic modifications, such as DNA methylation and histone modifications, and these processes play an important role in the regulation of gene expression for proteins like E-cadherin during EMT [22]. Histone methylation of the Lys9 and Lys27 residues of histone H3 (H3K9me2/3 and H3K27me3) represses gene expression; in contrast, histone acetylation of H3K4 (H3K4Ac) and H3K9 (H3K9Ac) is associated with gene activation [23].

Recently, G9a, a Snail-interacting protein, emerged as an important mediator of Snail-induced EMT. G9a, also called EHMT2 or KMT1C, is a major euchromatic methyltransferase, which mediates gene silencing in euchromatin by mono- and di-methylation of on histone 3 lysine 9 (H3K9) [24]. G9a can cooperate with other transcription factors to regulate gene expression [25], and is reportedly involved with important cancer-sustaining cellular activities such as cell proliferation, autophagy, EMT, metabolic changes, specific responses to hypoxia and cancer stemness [26–30]. Recent studies show that G9a is highly expressed in many types of malignant tissue, including HNSCC, when compared to paired normal tissue [31, 32]. However, it is still unknown whether G9a plays a role in HNSCC metastasis to lymph nodes.

Since cancer stem cells (CSCs) are capable of self-renew and proliferation, they are thought to initiate tumorigenesis, as well as tumor recurrence after treatment. In addition, CSCs are likely responsible for therapeutic resistance and metastasis in HNSCC. CD44, a CSC marker in HNSCC, plays a role in tumor metastasis [33]. CD44 is a cell-surface glycoprotein that functions as a receptor for hyaluronic acid and is involved in cell adhesion and migration [34]. In HNSCC, a subpopulation of CD44^+^ CSCs displays a phenotypic switch, and become either proliferative or migratory [35]. Recent studies suggest that EMT in cancer cells is a process crucial for the acquisition of “stemness”, the ability to become a CSC [36]. However, the molecular mechanisms underlying stemness and metastasis in HNSCC are obscure.

In this study, we investigated the role of G9a in the induction of EMT and CSC-like characteristics in HNSCC. We found that G9a, through interaction with Snail, is required for both metastasis to lymph nodes and TGF-β induced EMT in HNSCC. Our study provides a plausible mechanism for metastasis to lymph nodes and EMT in HNSCC.

## RESULTS

### Lost of E-cadherin is associated with metastasis and poor clinical outcome in HNSCC

**Figure 1:**
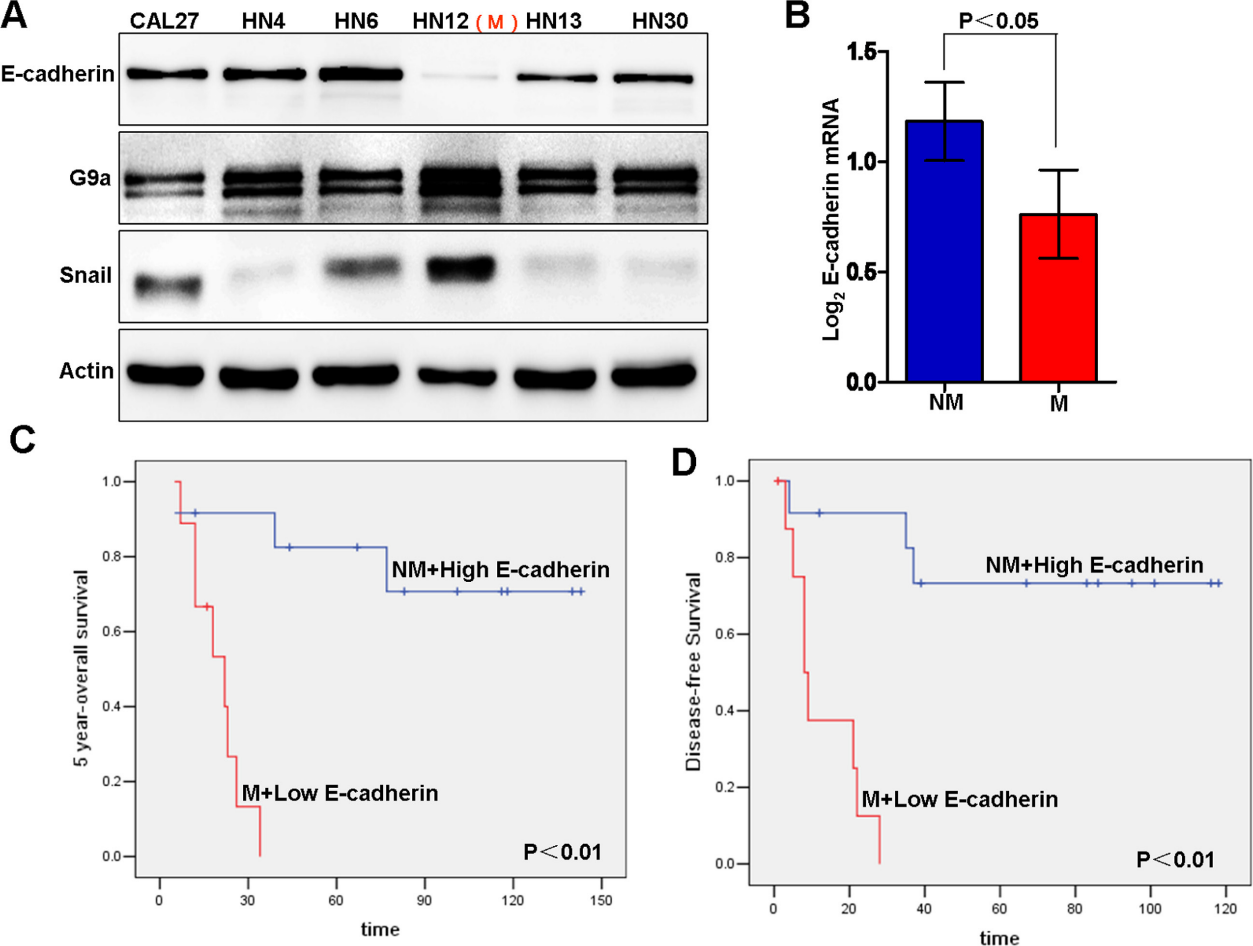
E-cadherin repression is associated with metastasis and poor clinical outcome in head and neck squamous cell carcinoma (HNSCC) **(A)** Western blot of E-cadherin, G9a and Snail using different HNSCC cell lines. “M” indicates the metastatic subtype of HN12 cells derived from a nodal metastasis. **(B)** E-cadherin mRNA levels and metastatic status of patients from an Oncomine dataset (Mean ± SD, *p* < 0.05). “NM” means “none-metastasic”; “M” means “metastasic”. **(C–D)** Kaplan-Meier survival curves demonstrate the 5-year survival analysis of combined metastasis status and E-cadherin expression level in HNSCC patients from an Oncomine dataset.

As loss of E-cadherin expression is a hallmarker of EMT, we first screened the expression level of E-cadherin in a panel of HNSCC cell lines. Immunoblot analysis showed a significant suppression of E-cadherin in HN12 as compared to other cell lines such as HN4 (Figure 1A). It was reported that HN12 and HN4 cells were derived from the same patient, but HN12 was a nodal metastatic subclone from HN4 cells [37]. This information suggests that loss of E-cadherin expression may be a crucial step for conversion from a non-metastatic (HN4) to metastatic (HN12) state in this HNSCC patient. To investigate the potential relationship between EMT and metastasis, we examined E-cadherin expression in HNSCC patients using a dataset from Oncomine, which includes 34 HNSCC and 4 normal uvula samples analyzed by Affymetrix U95A microarrays [38]. The analysis demonstrates that E-cadherin expression was higher in patients without metastasis whereas it was significantly lower in patients with metastasis (Figure 1B). Although the trend is consistent with our assumption, that repression of E-cadherin is associated with poor prognosis in HNSCC, the difference by Kaplan–Meier survival analysis was not statistically significant, possibly resulting from the limited number of cases available (Figure S1A and S1B). However, when the statistical analysis combined the metastatic status and E-cadherin expression level, the 5 year survival of patients with low E-cadherin expression and metastasis (M) was significantly shorter than survival with high E-cadherin and no metastasis (NM) (Figure 1C and 1D). Taken together, these results suggest that loss of E-cadherin expression correlates with HNSCC metastasis, a negative prognostic indicator in patients with HNSCC.

### EMT plays a key role in metastasis to lymph nodes of HNSCC

**Figure 2:**
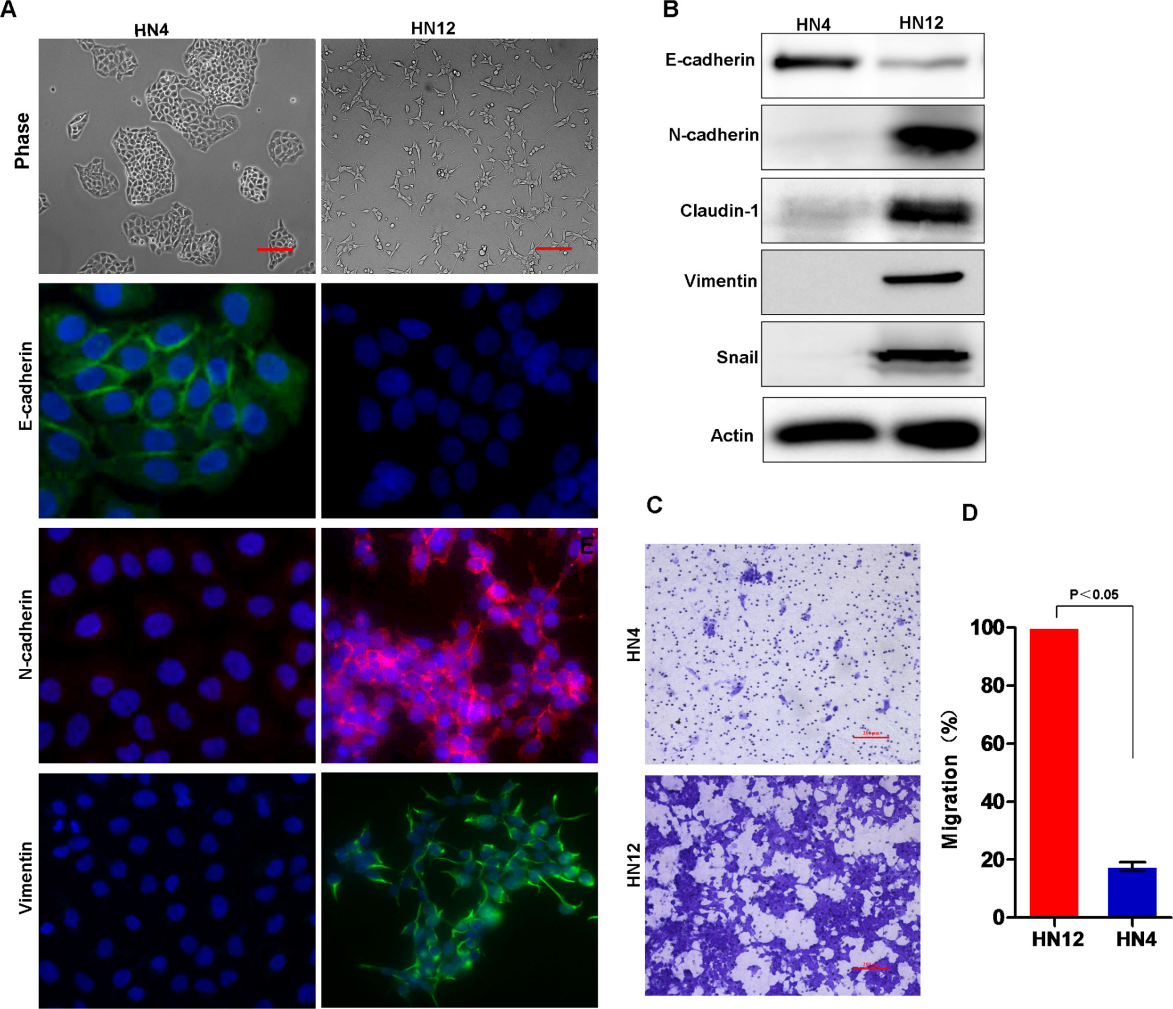
Lymph node metastatic HNSCC cells exhibit EMT characters **(A)** Morphology and staining for E-cadherin, N-cadherin and vimentin in HN-4 and HN12 cells. Scale bar = 200 μm. **(B)** Western blot analysis of E-cadherin, N-cadherin, Claudin-1, vimentin and Snail protein levels in HN4 and HN12 cell lines. **(C)** The transwell migration assay identified the migration capability of HN4 and HN12 cells with representative images shown. Scale bar = 200 μm. **(D)** Graph demonstrates the mean ± SD for the percent of migrated cells from 3 separate experiments.

To investigate the molecular mechanisms involved in HNSCC metastasis to lymph nodes, we selected HN4 and HN12 as a paired cell line for further characterization, since HN12 and HN4 cells were derived from the same patient, with HN12 a nodal metastatic subclone from the HN4 primary tumor [37]. HN4 cells exhibit the typical polygonal morphology for epithelial cells (Figure 2A). Immunofluorescent analysis showed high expression levels of the epithelial marker E-cadherin and low levels of mesenchymal markers N-cadherin and vimentin in HN4 cells (Figure 2A). In contrast, HN12 cells were scattered throughout the plate surface, displayed a fibroblast-like morphology, and expressed low levels of E-cadherin and high levels of the N-cadherin and vimentin (Figure 2A and 2B). Immunoblot analysis confirmed the molecular features of these two cell lines (Figure 2B). Next, we examined the migratory capabilities of HN4 and HN12 cells, an EMT-associated biological activity, using a transwell migration assay. HN12 cells exhibited a significantly higher motility than did the HN4 cells (Figure 2C and 2D). Taken together, these results indicate that HN12 cells gain EMT-related molecular and functional phenotypic changes relative to their companion HN4 cells. Thus, EMT may play a key role in metastasis to lymph nodes in HNSCC.

### G9a interacts with snail and binds to the promoter of E-cadherin as a complex

**Figure 3:**
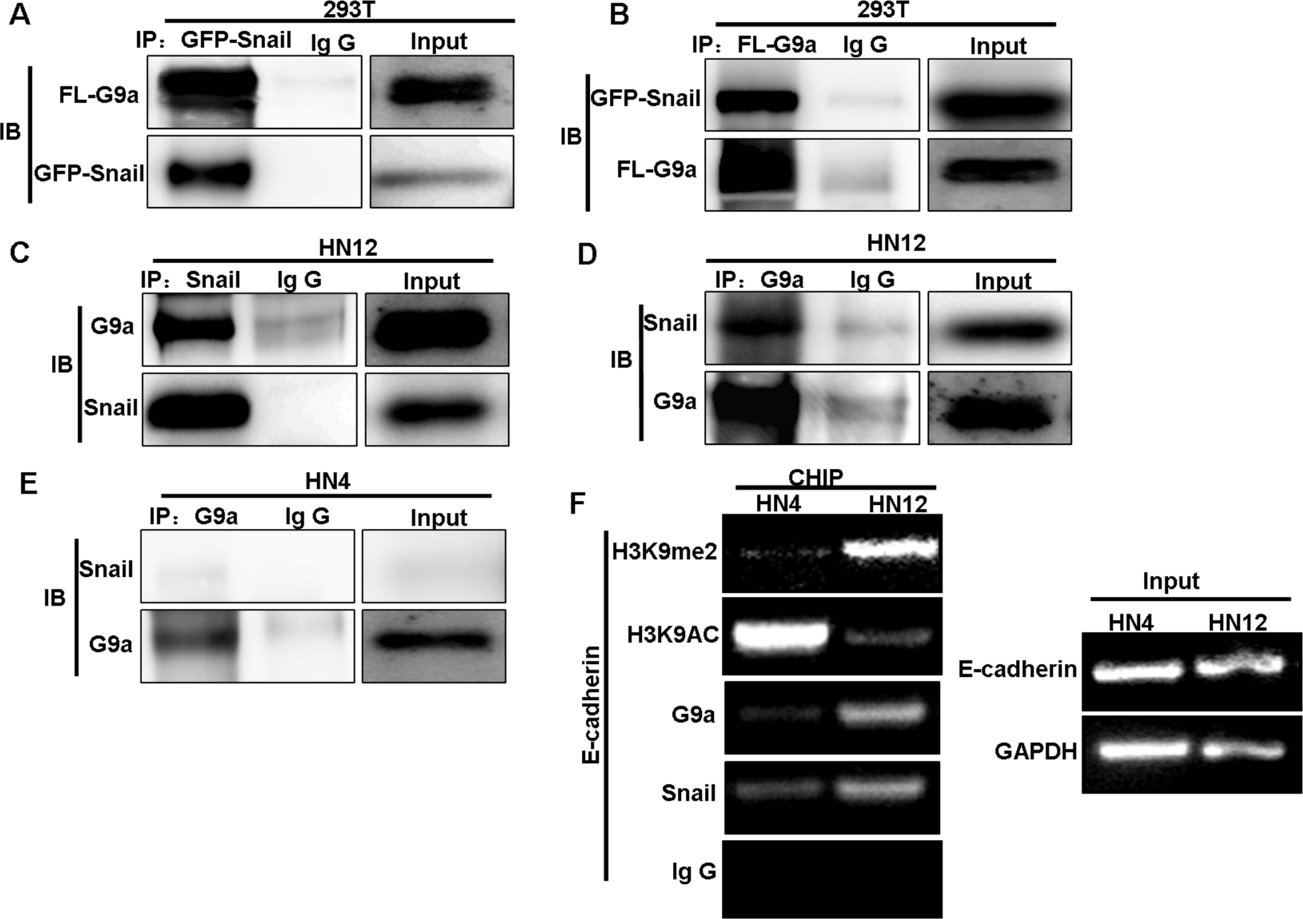
G9a interacts with Snail and binds to the E-cadherin promoter **(A–B)** 293T cells were transiently transfected with Flag-tagged G9a GFP-tagged Snail plasmids. Western blot analysis of cell extracts immunoprecipitated (IP) with either Flag or GFP antibodies, and their associated G9a, and Snail proteins. **(C, D, E)** Endogenous Snail and G9a were immunoprecipitated from HN12 and HN4 cells, and examined by Western blot. **(F)** ChIP analysis demonstrates the association of G9a, Snail, and the level of H3K9me2 and H3K9 acetylation at the E-cadherin promoter in HN4 and HN12 cell lines.

G9a is a critical component of Snail-induced repression of E-cadherin in human breast cancer [27], but its involvement in lymph node metastasis in HNSCC is unknown. To identify a relationship between E-cadherin and G9a, we analyzed the expression of E-cadherin and G9a from Oncomine data sets, which contain 34 HNSCC tumor samples (Figure S1C). We did not find any correlation in the expression of E-cadherin with G9a at the mRNA level in this gene expression data set. Similarly, examination of E-cadherin and G9a protein levels in a panel of HNSCC cell lines did not reveal any correlation in protein expression (Figure 1A). To explore the potential involvement of G9a, we examined the interaction of G9a with Snail by co-immunoprecipitation (Co-IP) following transient transfection of HEK293T cells with Flag-tagged G9a and GFP-tagged Snail. The analysis confirmed that G9a and Snail interact to form a complex, since immunoprecipitation of either G9a or Snail revealed the other molecule (Figure 3A and 3B). Importantly, only the metastatic HNSCC cell line, HN12, showed a physical interaction between endogenous Snail and G9a (Figure 3C–3D); this interaction was not detected in the non-metastatic HNSCC cell line HN4 (Figure 3E). These findings suggest that the interaction between G9a and Snail may be crucial for the promotion of metastatic features in HN12 cells.

To determine if the G9a-Snail complex influences E-cadherin repression, we performed chromatin immunoprecipitation (ChIP) analysis on the E-cadherin promoter of HN4 and HN12 cells. We found dramatically higher levels of H3K9me2, an indicator of gene silencing, at the E-cadherin promoter of HN12 cells when compared to levels found in HN4 cells (Figure 3F). In contrast, H3K9 acetylation, an indicator of gene expression, was much lower at the E-cadherin promoter in HN12 cells than that in HN4 cells (Figure 3F). The elevated levels of H3K9me2 at the E-cadherin promoter are likely due to formation of a Snail–G9a complex because the occupancy of Snail and G9a at the E-cadherin promoter was significantly higher in HN12 cells than in HN4 cells (Figure 3F). Thus, in metastatic HN12 cells, G9a forms a complex with Snail and binds to the E-cadherin promoter, which results in H3K9me2 and eventually DNA methylation; these events increase the potential for EMT and metastasis to lymph nodes in this HNSCC cell line.

### G9a-mediated H3K9 methylation is required for TGF-β-induced EMT in HNSCC

**Figure 4:**
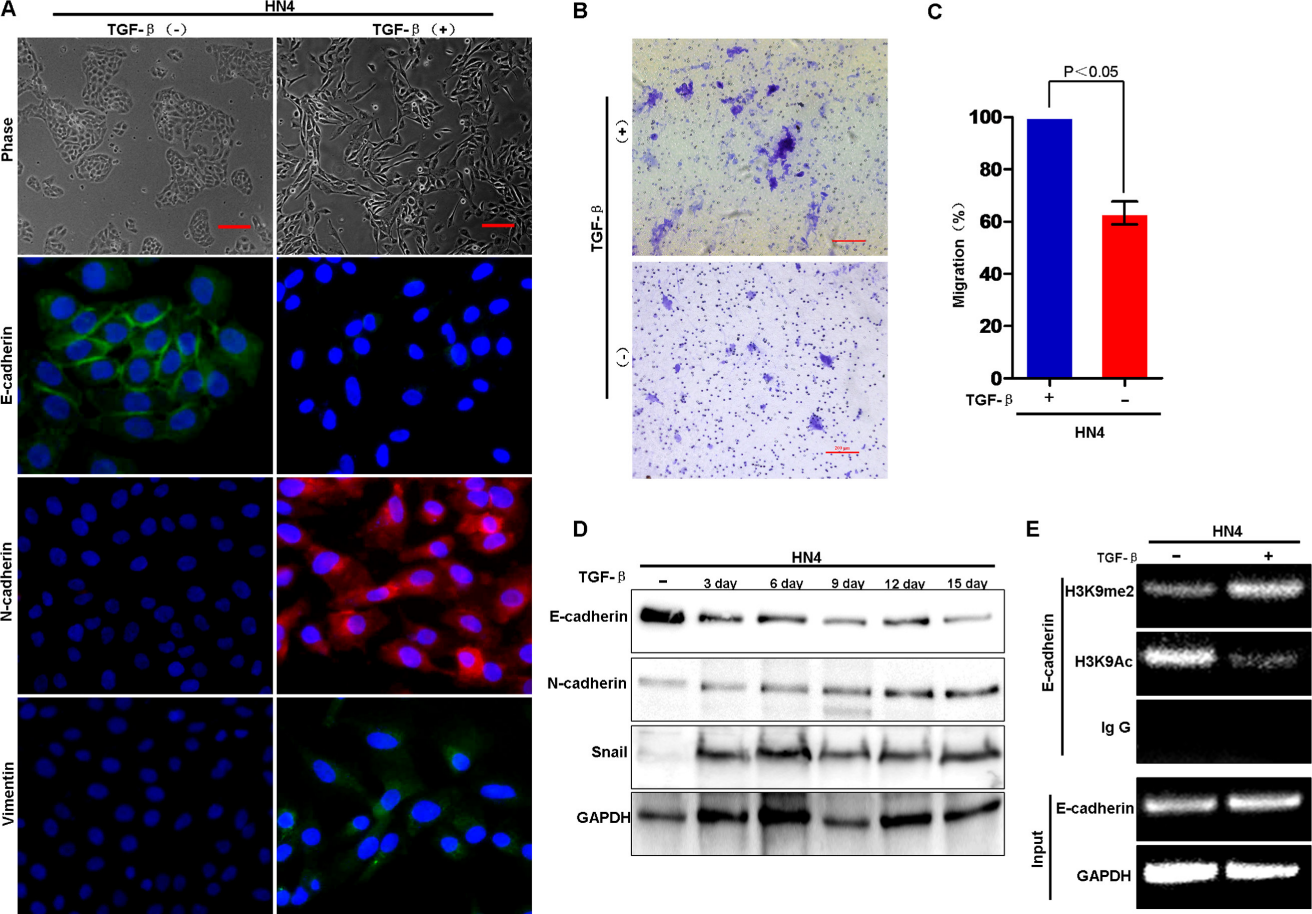
H3K9 methylation at the E-cadherin promoter is associated with TGF-β–induced EMT in HNSCC **(A)** HN4 cells were treated with TGF-β1 (5 ng/ml) for 3, 6, 9, 12 and 15 days, respectively; morphologic changes associated with EMT at 12 days are shown in the phase contrast images. Immunofluorescence (IF) staining for E-cadherin, N-cadherin and vimentin is presented with nuclei stained with DAPI (blue). Scale bar = 200 μm. **(B)** Migration of HN4 cells exposed to TGF-β (5 ng/ml) for 6 days before assay with representative images shown. Scale bar = 200 μm. **(C)** Graph demonstrates the mean ± SD percent of migrated cells from 3 separate experiments. **(D)** Western blot analysis of E-cadherin, N-cadherin and Snail in HN4 cells treated with TGF-β (5 ng/ml) for the indicated time periods. **(E)** ChIP analysis of H3K9me2 and H3K9Ac at the E-cadherin promoter of HN4 cells treated with TGF-β (5 ng/ml).

**Figure 5:**
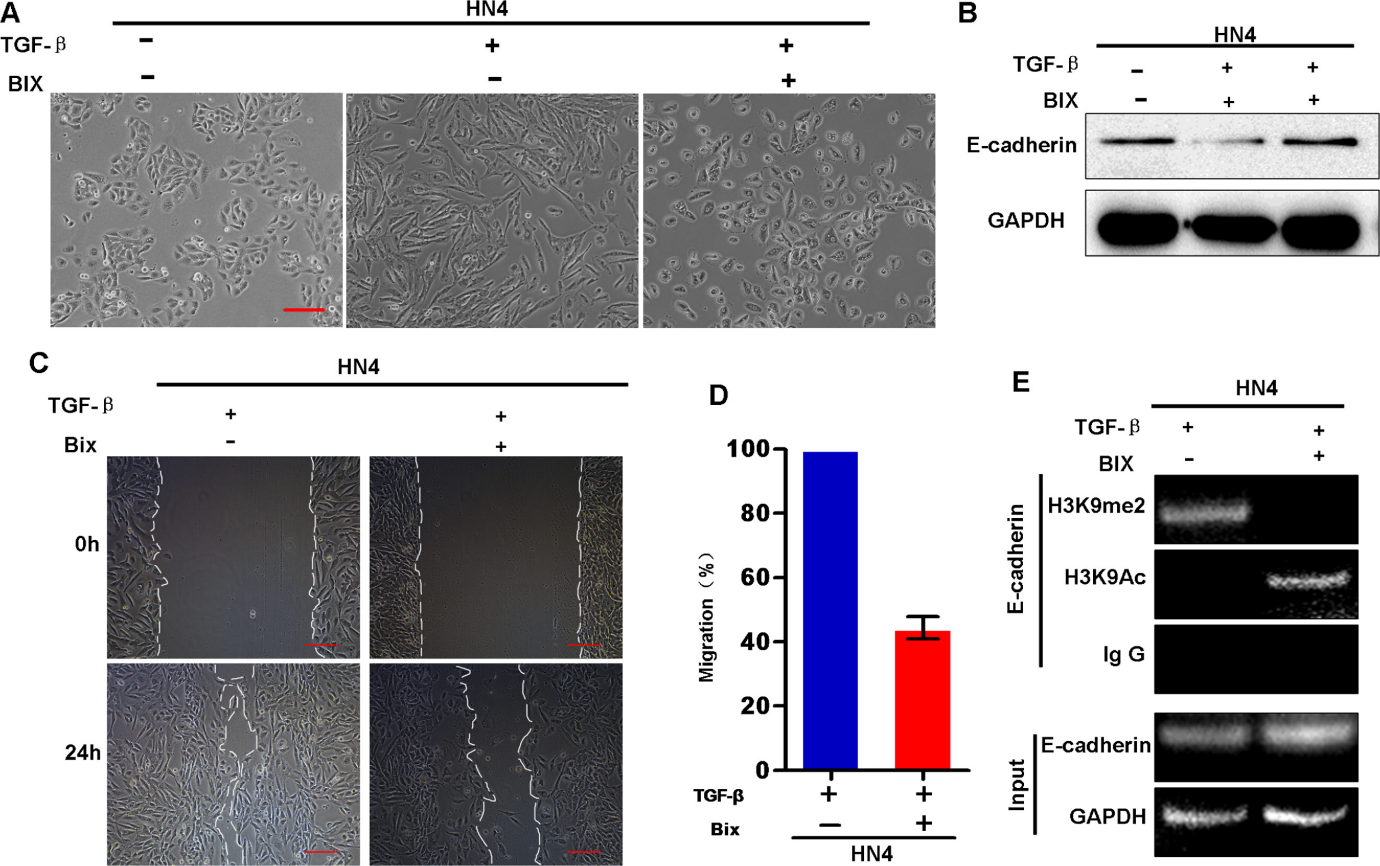
G9a inhibitor BIX01294 reverses TGF-β–induced EMT in HNSCC cells **(A)** Morphologic changes of HN4 cells treated with or without BIX01294 (BIX: 2.5 μM) followed by treatment with TGF-β1 (5 ng/ml) for 5 days. **(B)** Western blot analysis of E-cadherin protein levels of BIX-treated HN4 cells with and without additional TGFβ treatment. **(C)** Wound healing and migratory analysis of HN4 cells treated with or without BIX01294 followed by TGF-β1 (5 ng/ml) treatment. Scale bar = 200 μm. **(D)** Graph demonstrates the migratory ability of HN4 cells (mean ± SD) treated with or without BIX01294 followed by TGF-β1 (5 ng/ml) treatment from 3 separate experiments. **(E)** ChIP analysis of H3K9me2 and H3K9 acetylation at the E-cadherin promoter of BIX-treated HN4 cells with and without additional TGF-β treatment.

It appears that HN12 cells are ‘locked’ in the mesenchymal state since the E-cadherin promoter is methylated in these cells; this situation makes it difficult to study the initial and dynamic events of EMT in HNSCC. To overcome this technical issue, we investigated the mechanisms underlying histone modification and DNA methylation during TGF-β-induced EMT in HN4 cells. We found that, TGF-β treatment (5 ng/ml) induced EMT in HN4 cell as characterized by acquisition of fibroblastic mesenchymal morphology (Figure 4A), downregulation of the epithelial marker E-cadherin, and upregulation of mesenchymal markers N-cadherin and vimentin (Figure 4A and 4D). The transwell migration assay showed that TGF-β treatment significantly increased the number of migrating cells, suggesting that TGF-β increased the motility of HN4 cells (Figure 4B and 4C). Moreover, ChIP analysis showed that TGF-β treatment significantly increased H3K9me2, and decreased H3K9 acetylation at the E-cadherin promoter in HN4 cells (Figure 4E). Taken together, the results suggest that H3K9me2 is involved in TGF-β-induced EMT in HNSCC. Because the methyltransferase activity of G9a is specific, the elevated level of H3K9me2 on the E-cadherin promoter implies that G9a might be involved in regulation of E-cadherin expression. To determine if the G9a-Snail complex in the E-cadherin promoter is essential for E-cadherin repression in HNSCC, we examined the effects of an inhibitor of G9a, BIX01294, on the H3K9me2 methyltransferase activity in the TGF-β-induced EMT model. BIX01294 treatment reversed the EMT phenotype induced by TGF-β treatment: the fibroblastic mesenchymal morphology reverted to the polygonal epithelial morphology (Figure 5A). Moreover, BIX01294 treatment upregulated E-cadherin expression (Figure 5B) and reduced the wound healing activity in the TGF-β-induced EMT model (Figure 5C–5D). Consistently, ChIP analysis showed that BIX treatment significantly decreased the level of H3K9me2 and increased the level of H3K9Ac on the E-cadherin promoter (Figure 5E), which accounts for the upregulated E-cadherin in BIX-treated cells. Thus, G9a is essential for the formation of H3K9me2 on the E-cadherin promoter, which mediates TGF-β-induced EMT in HNSCC cells.

**Figure 6:**
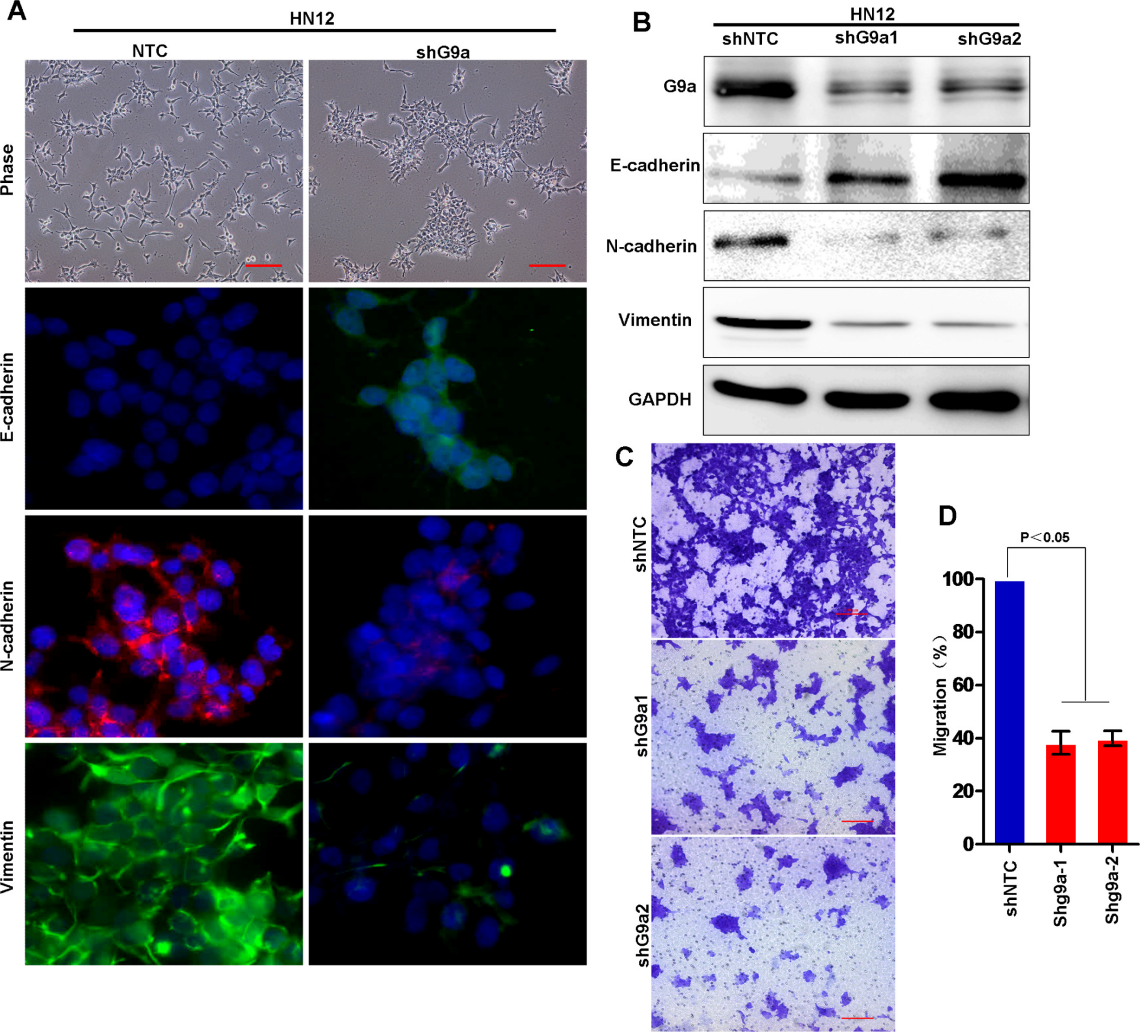
Knockdown of G9a expression inhibits cell migration in HNSCC **(A)** Morphologic changes in stably transfected HN12 cells with knockdown of G9a and control HN12 cells by phase contrast microscopy. Immunofluorescence (IF) staining for E-cadherin, N-cadherin and vimentin is presented with nuclei stained with DAPI (blue). Scale bar = 200 μm. **(B)** Western blot analysis of E-cadherin, N-cadherin and vimentin in HN12 cells stably expressing control vector or G9a shRNA. **(C)** Migration of HN12 cells stably expressing control vector or G9a shRNA analyzed using the transwell migration assay. Scale bar = 200 μm. **(D)** Graph demonstrates the mean ± SD percent migrated cells in HN12 vector control and shG9a cells from 3 separate experiments.

To confirm this observation, we established stable shRNA-G9a transfectants of HN12 cells with knockdown of G9a expression. The knockdown efficiency of endogenous G9a by shRNA was about 80% (Figure 6B). Knockdown of G9a restored E-cadherin expression and dramatically downregulated N-cadherin and vimentin in HN12 cells (Figure 6A and 6B). In addition, knockdown of G9a greatly inhibited the motility and migration of HN12 cells (Figure 6C and 6D). Taken together, these results strongly support the assertion that G9a is essential for repression of E-cadherin, with subsequent EMT and metastasis to lymph nodes in HNSCC.

### Induction of CSC characters is associated with EMT in HNSCC

**Figure 7:**
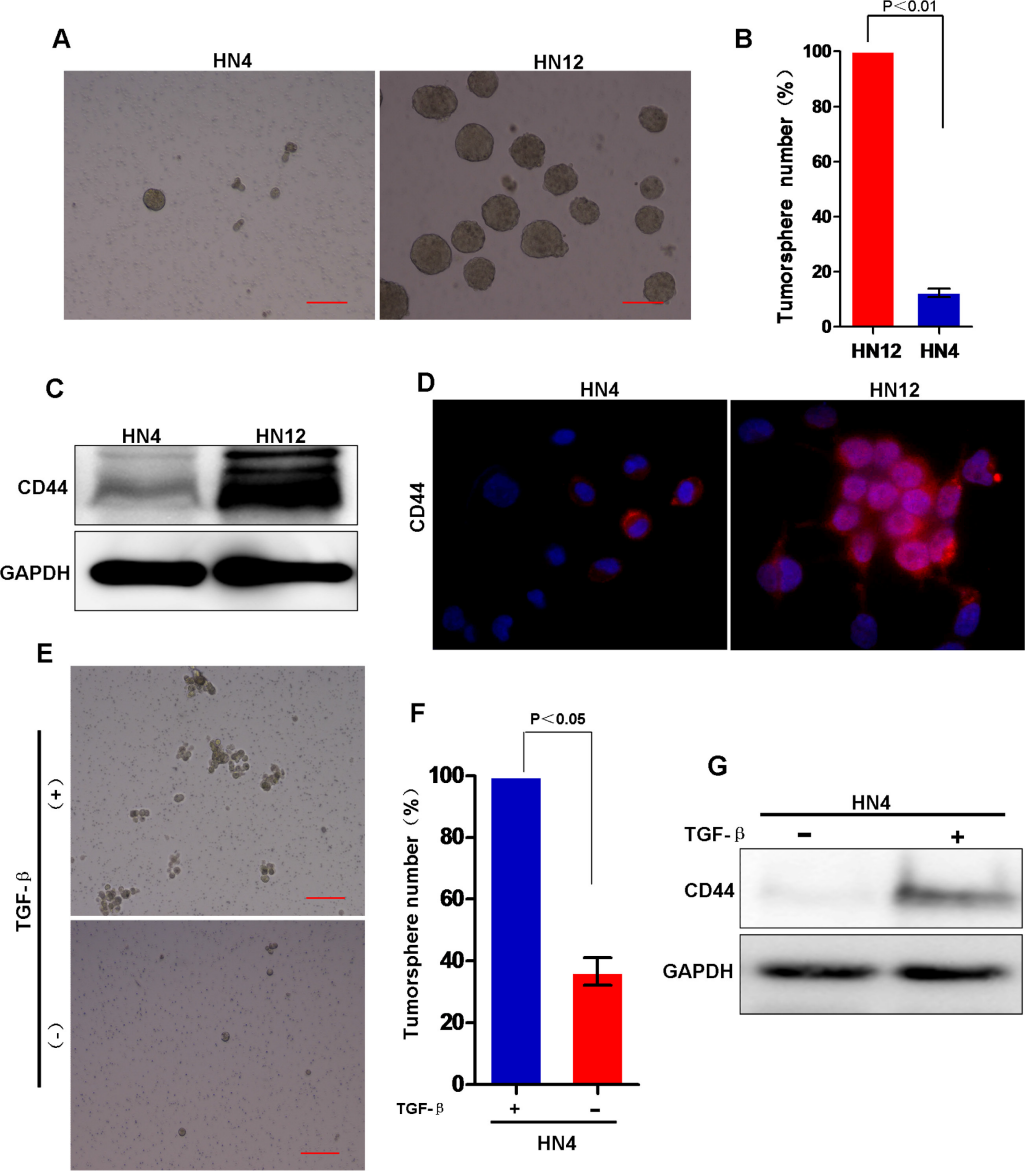
Stem cell-like characters is involved in the EMT process in HNSCC **(A)** Representative image of tumorsphere formation in HN4 and HN12 cells. Scale bar = 200 μm. **(B)** Measurement of tumorsphere formation (mean ± SD from 3 separate experiments) in HN12 and HN4 cells. **(C)** Western blot analysis of CD44 in HN4 and HN12 cells. **(D)** Images are immunofluorescent staining of CD44 in HN4 and HN12 cells. **(E)** Representative image of tumorsphere formation in HN4 cells with or without TGF-β treatment. **(F)** Measurement of tumorsphere formation (mean ± SD from 3 separate experiments) in HN4 cells with or without TGF-β treatment. **(G)** Western blot analysis of CD44 in HN4 cells with or without TGF-β treatment.

Recent studies suggest that EMT is involved in acquisition of CSC properties, such as an increased ability to form tumorspheres, and expression of stem cell-like markers [36]. To determine whether the EMT status of HNSCC enhances CSC characteristics, especially in EMT-related metastasis to lymph nodes, we performed a series of experiments with the HN4 and HN12 cell lines. We found tumorsphere formation significantly increased in HN12 cells when compared with formation in HN4 cells (Figure 7A and 7B). Next, we examined the CSC marker CD44 in HNSCC cells by Western blot analysis [33]; CD44 expression increased in HN12 cells when compared with expression levels in HN4 cells (Figure 7C and 7D). Consistently, FACS analyses also showed CD44 positive cells significantly increased in HN12 cells when compared with levels in HN4 cells (Figure S2A–S2B). Moreover, colony formation in soft agar was significantly greater in HN12 cells than in HN4 cells (Figure S2C and S2D). Consistent with these observations, TGF-β treatment of HN4 cells increased the tumorsphere-formation and CD44 expression (Figure 7E and 7G), suggesting that TGF-β-induced EMT is associated with increased CSC characters in HNSCC. Taken together, these observations suggest that induction of EMT in HNSCC generates CSC-like cells.

### G9a is essential for EMT-induced CSC characters in HNSCC

**Figure 8:**
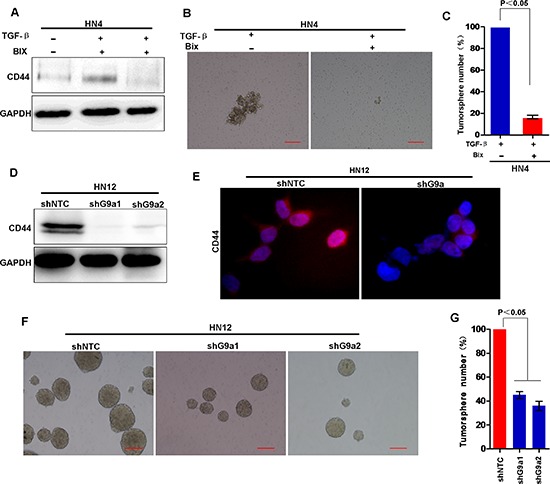
Knockdown of G9a inhibit tumorsphere formation and suppress the expression of CD44 **(A)** Western blot analysis of CD44 expression in HN4 cells treated without or with BIX (2.5 μM) followed by treatment with TGF-β (5 ng/ml). **(B)** Representative image of tumorsphere formation in HN4 cells treated without or with BIX (2.5 μM) followed by treatment with TGF-β (5 ng/ml). **(C)** The tumorsphere formation was measured in HN4 cells treated without or with BIX (2.5 μM) followed by treatment with TGF-β (5 ng/ml). Mean ± SD from 3 separate experiments. **(D)** Western blot analysis of CD44 expression in HN12 cells stably expressing control vector or G9a shRNA. **(E)** Image of immunofluorescence staining for CD44 expression. **(F)** Image of tumorsphere formation in HN12 cells stably expressing control vector or G9a shRNA. Scale bar = 200 μm. **(G)** Graph demonstrates the mean ± SD for tumorsphere number in HN12 cells stably expressing control vector or G9a shRNA from 3 separate experiments.

Since G9a is essential for TGF-β-induced EMT, and EMT induces CSC-like characteristics, we asked whether G9a is essential for induction of CSC-like characteristics in HNSCC. TGF-β treatment induced expression of the CD44 marker in HN4 cells (Figure 8A). However, addition of a G9a inhibitor BIX01294 suppressed CD44 expression, suggesting that a functional G9a is required for TGF-β-induced CD44 expression in these cells. Consistently, BIX01294 also suppressed TGF-β-induced tumorsphere-formation (Figure 8B and 8C). These results suggest that a functional G9a is required for TGF-β-induced CSC-like characters. To determine if G9a is essential for maintenance of CSC-like characteristics, we examined and compared the biochemical and biological characteristics of stable shRNA-G9a transfectants (knockdown of G9a expression) with HN12 cells. Knockdown of G9a expression significantly suppressed CD44 expression when levels were compared to control HN12 cells, suggesting that G9a is required for CD44 expression (Figure 8D, 8E and Figure S3). Moreover, knockdown of G9a also suppressed tumorsphere formation in the transfected cells compared with formation in control HN12 cells (Figure 8F and 8G). Taken together, these observations demonstrate that G9a is essential for induction and maintenance of EMT-related CSC-like characteristics in HNSCC.

## DISCUSSION

In this study, we show that repressed E-cadherin expression correlates with cancer metastasis and poor prognosis in HNSCC. We found that G9a is essential for Snail-induced repression of E-cadherin and EMT in HNSCC cells. Furthermore, we demonstrated that G9a is required for induction of CSC-like characters in HNSCCs. Thus, the Snail-G9a axis is a vital component of metastatic HNSCC, and provides a potential therapeutic target for this disease.

Metastasis, the major cause of cancer deaths, is a multistep process consisting of four distinct steps: invasion, intravasation, extravasation and metastatic colonization. The initiation of tumor cell migration is a prerequisite for the metastatic cascade. EMT, considered the most important step in initiating cancer metastasis, associates with decreased or complete loss of E-cadherin expression in HNSCC metastatic to lymph nodes [19]. These conclusions were obtained from various analyses including IHC [39, 40] and mRNA array (Figure 1). Many transcriptional repressors, such as Snail, Slug, Twist, ZEB1, ZEB2, have been implicated in the regulation of EMT for a variety of cancers, including breast cancer, colon cancer, liver and HNSCC [27, 41–44]. For example, Slug, Twist, Snail and ZEB1 are important in E-cadherin repression, and thus regulating EMT, especially in breast cancer. In this study, we have shown that the Snail-G9a axis plays a critical role in mediating EMT in HNSCC. Our observation is supported by Mendelsohn [45] who used IHC to demonstrated an upregulated Snail expression in HNSCC, along with a correlation between Snail levels and metastasis in this disease. However, other pathways may contribute to EMT in HNSCC. For example, Slug and Twist were upregulated in HNSCC metastatic sites, and both promote EMT-mediated metastasis. However, the molecular mechanisms regulating these proteins require further investigation.

The Snail-G9a complex is bound to the E-cadherin promoter in HN12 cells but not in HN4 cells. The expression level of G9a is similar in HN12 and HN4 cells, but Snail expression is high in HN12 cells, and barely detectable in HN4 cells. This observation strongly supports the hypothesis that Snail level is the key factor for EMT induction. However, both Snail-induced and TGF-β-induced repression of E-cadherin require G9a since knockdown of G9a by shRNA or suppression of G9a activity by G9a inhibitor BIX reverse EMT markers and inhibit HNSCC cell migration. Thus, G9a is a major component mediating Snail-induced effects, at least in the HNSCC cells tested.

G9a is a key methyltransferase responsible for mono- and di-methylation of lysine 9 on histone 3 (H3K9) of euchromatin and facultative heterochromatin [24]. Recent studies demonstrate that G9a is vital for Snail-mediated EMT in human breast cancer [27]. Our study extends these findings, confirms the importance of G9a epigenetic modification on the E-cadherin promoter in HNSCC, and reinforces the view that EMT is an epigenetic event. In addition to facilitating Snail functions, G9a also interacts with other transcription factors, including Sharp-1, Gfi1, NF-κB, CDP, and REST. In a manner similar to the Snail-G9a complex, these proteins complex with and are recruited to distinct target promoters [46–50]. For example, G9a interacts with Sharp-1 and enhances Sharp-1–dependent repression of MyoD in a methyltransferase activity-dependent manner [46]. While primarily thought to generate H3K9me2 for transcriptional repression, G9a plays a more complicated role in regulating the transcriptional activation of other genes. For example, G9a can be recruited to and activate the β globin locus in an NF-E2/p45-dependent manner [51]. Similarly, G9a can act as a co-activator together with GRIP1, CARM1 and p300 for nuclear receptors in a methyltransferase-independent manner [58]. For example, recruited G9a is bound to the glucocorticoid receptor (GR) binding site, and functions as a scaffold for the recruitment of p300 and CARM1 to activate gene expression. In addition, Runx2 recruits G9a to MMP-9, CSF-2, SDF-1 and CST7 promoters to activate their expression in a methyltransferase activity-independent manner [52]. Together these studies demonstrate that G9a is a multifunctional regulator of gene expression that can function either as a repressor or as an activator. We found that on one hand, G9a interacts with Snail and plays a “repressor” role for E-cadherin expression, whereas knockdown of G9a blocked CD44 expression, suggesting that G9a acts as an “activator” of CD44 expression. However, it is unclear whether the regulation of CD44 expression by G9a is direct, that is, regulated at the promoter, or indirect and through other mechanisms.

Recent and transformative studies show that EMT is capable of introducing stem-like properties in epithelial cells [36]. CSC is a subpopulation of cells with self-renewing capacities, believed to confer resistance to chemo- and radio-therapy and is responsible for tumor maintenance and metastasis [53]. The CSCs hypothesis suggests that tumors can arise from stem or progenitor cells. Importantly, many studies show that EMT can confer tumor cells with stem cell-like characteristics [36, 54, 55]. Proposed mechanisms for the EMT-induced stem-like properties of cancer cells vary. For example, a report indicates that ZEB1 inhibits the expression of the miR-200 family, resulting in upregulation of polycomb protein Bmi1 and induction of stemness in pancreatic cancer [59]. In HNSCC, one study showed that the EMT inducer Twist1 directly activates Bmi1 transcription, and that Twist and Bmi1 act cooperatively to induce EMT and stemness [56]. In this study, we found CSC-related characteristics induced in both lymph node-related EMT (HN12 cells) and TGF-β-induced EMT (HN4 cells). These data suggest that there is a biological link between CSC and EMT in HNSCC. As expected, inhibition of G9a by the inhibitor BIX not only repressed CD44 expression in HN12 cells and TGF-β-induced EMT in HN4 cells, but also suppressed the tumorsphere-formation. Thus, G9a is critical for both induction of EMT and maintenance of stem cell-like properties.

Because EMT influences both CSC properties and metastatic activity, targeting the EMT/CSC phenotype becomes an attractive strategy for the treatment of metastasis and tumor reoccurrence. Thus, targeting Snail–G9a axis may represent a novel approach for treating and/or preventing metastatic HNSCC. Many cancers show an upregulation of both Snail and G9a including HNSCC [31, 32, 57]. Because either knockdown of G9a expression or suppression by BIX01294 inhibits migration and tumorsphere formation, targeting Snail and G9a, or their interaction, may repress EMT and the stem cell-like properties in HNSCC. Further investigation in targeting the Snail-G9a axis with animal tumor models is desirable.

In summary, mediation of Snail-induced EMT and repression of E-cadherin in HNSCC cells requires a functional G9a protein. Moreover, a link exists between EMT and CSC in HNSCC. Thus, targeting the G9a-Snail axis may represent an attractive strategy to target metastatic and tumor recurrence in HNSCC.

## MATERIALS AND METHODS

### Plasmids and antibodies

Preparation of the expression plasmid for human Snail was described previously [15]. Antibodies against actin and GAPDH were from Santa Cruz Biotechnology (Santa Cruz, CA). Antibodies for E-cadherin, N-cadherin, vimentin, CD44, Snail, and Claudin-1 were from Cell Signaling Technology Inc. (Beverley, MA,). G9a, H3K9me2, and H3k9Ac antibodies were from Abcam (Cambridge, MA).

### Cell cultures

The head and neck squamous cell carcinoma-derived cell lines HN4, HN6, HN12, HN13, HN30 were kindly provided by the University of Maryland, School of Dentistry. HN4 cancer cells, derived from a primary squamous cell carcinoma of the tongue, have an epithelial phenotype and low invasive capacity. HN12 cancer cells exhibit a mesenchymal phenotype and have high invasive capacity; they were derived from a nodal metastasis in the patient from whom the HN-4 cells originated [37, 60]. The CAL27 cell line was purchased from the American Type Culture Collection (ATCC; Manassas, VA). All cells were cultured in Dulbecco's modified Eagle's medium (DMEM; GIBCO, CA) supplemented with 10% FBS, 1% glutamine, and 1% penicillin–streptomycin, and maintained in a humidified atmosphere of 5% CO_2_ at 37°C.

Establishment of HN12 cells with knockdown of G9a expression was accomplished by transfection with G9a shRNA (Sigma-Aldrich, St. Louis, MO), and stable clones selected with puromycin (500 ng/ml) for 4 weeks.

### Immunostaining, immunoprecipitation, and immunoblotting

Experimental protocols for immunoprecipitation and immunoblotting follow those previously described [61]. For immunofluorescence staining, cultured cells rinsed three times with PBS and fixed with 3.7% formaldehyde were then permeabilized with 0.1% Triton X-100. After blocking in 1% BSA for 1 hour, cells were incubated with the primary antibody in a moist, 4ºC chamber overnight, washed and then incubated for 1 hour with Alexa Fluor 488 (in the dark), or 594 donkey anti-rabbit IgG (H + L) antibody (Invitrogen, Grand Island, NY) at room temperature. Washed cells (3X; PBS containing 0.02% Tween 20), stained by mounting onto a slide with aqueous mounting medium containing 0.5 mg/ml 40–6-diamidino-2-phenylindole, were examined with a fluorescence microscope (Nikon E800) at 400 × magnification.

### Flow cytometry analysis

Flow cytometry was performed as described previously [62]. Trypsinized cells washed twice with PBS were fixed. Cells stained with antibodies to CD44 or an IgG isotype (Santa Cruz) and labeled with Alexa 488-conjugated secondary antibody were subjected to flow cytometric analysis.

### Chromatin immunoprecipitation (ChIP)

ChIP analyses were performed using the Imprint ChIP Kit (Sigma–Aldrich) as described previously [27]. Briefly, cells crosslinked with formaldehyde at room temperature, were lysed with L1buffer (50 mM Tris, 2 mM EDTA, 0.1% IGEPAL, 10% glycerol, 1 mM dithiothreitol, 1 mM phenylmethylsulfonyl fluoride and protease inhibitor mixture, pH 8.0) on ice. After centrifugation, the nuclear pellet was re-suspended in ChIP lysis buffer (1% SDS, 10 mM EDTA, 50 mM Tris and protease inhibitor mixture, pH 8.0), and sonicated. Following an overnight incubation with antibody, a 50% slurry of protein A-agarose/salmon sperm DNA was added for 3 h. Bound DNA–protein complexes were eluted and crosslinks released after a series of washes. PCR analysis used the purified DNA resuspended in TE buffer (10 mM Tris-HCl and 1 mM EDTA, pH 8.0).

### Migration and wound healing assay

Experiments were performed as described previously [27]. For the migration assay, cells (5 × 10^5^) were seeded onto the upper chamber in 200 μL of serum-free medium; the lower compartment was filled with 0.6 mL of DMEM media supplemented with 10% of FBS. After a 24 h incubation, migrated cells on the lower surface of the filter were fixed and stained using propidium iodide; cells on the upper side were removed using a rubber scraper. Fluorescent images were obtained; reported data are counts of migrated cells with experiments performed in triplicate.

### Tumorsphere culture

The experimental protocol for tumorsphere formation was described previously [62]. Single-cell suspensions were prepared at a density of 4,000 cells per milliliter and seeded into six-well plates (2.0 mL per plate) coated with 1.2% poly-Hema (Sigma-Aldrich). Suspension cultures continued for 1–2 weeks until tumorspheres formed. Tumorspheres with diameter > 50 μM were counted. Recorded data are colony number obtained from 10 separate views using a microscope. Experiments were repeated three times with duplication in each experiment.

### Soft-agar and tumorigenesis assays

Soft-agar assays were performed as described previously [63]. Cells were suspended in 0.2 mL of Matrigel (Collaborative Biomedical Products, Becton Dickinson Labware, Bedford, MA) diluted 1:1(vol/vol) with growth medium supplemented with 10% FBS. Cell suspensions were then placed on top of a previously cast semisolid layer of 0.2 mL of 1% low–melting agarose in growth medium in each well of a 24-well plate. Colonies formed over a 2 wk period at 37°C in a humidified CO_2_ incubator. Colonies in four microscopic fields were then counted using an inverted microscope at 40× magnification and photographed. Reported data are the means and standard error from two independent experiments performed, in triplicate.

### Statistical analysis

Data analysis used SPSS (Statistic Package for Social Sciences) 13.0 for Windows (SPSS Inc., Chicago, IL, USA). Unpaired Student's *t*-tests or U-Mann Whitney tests determined statistical significance between groups with *p* values < 0.05 considered significant.

## SUPPLEMENTARY FIGURES


