# Supplementary material for: G9a is essential for EMT-mediated metastasis and maintenance of cancer stem cell-like characters in head and neck squamous cell carcinoma
Source: Oncotarget. 2015 Jan 29;6(9):6887–901. doi: 10.18632/oncotarget.3159 (PMC4466657; doi:10.18632/oncotarget.3159)
Supplement: Supplementary file 1 [file oncotarget-06-6887-s001.pdf]

SUPPLEMENTARY FIGURES

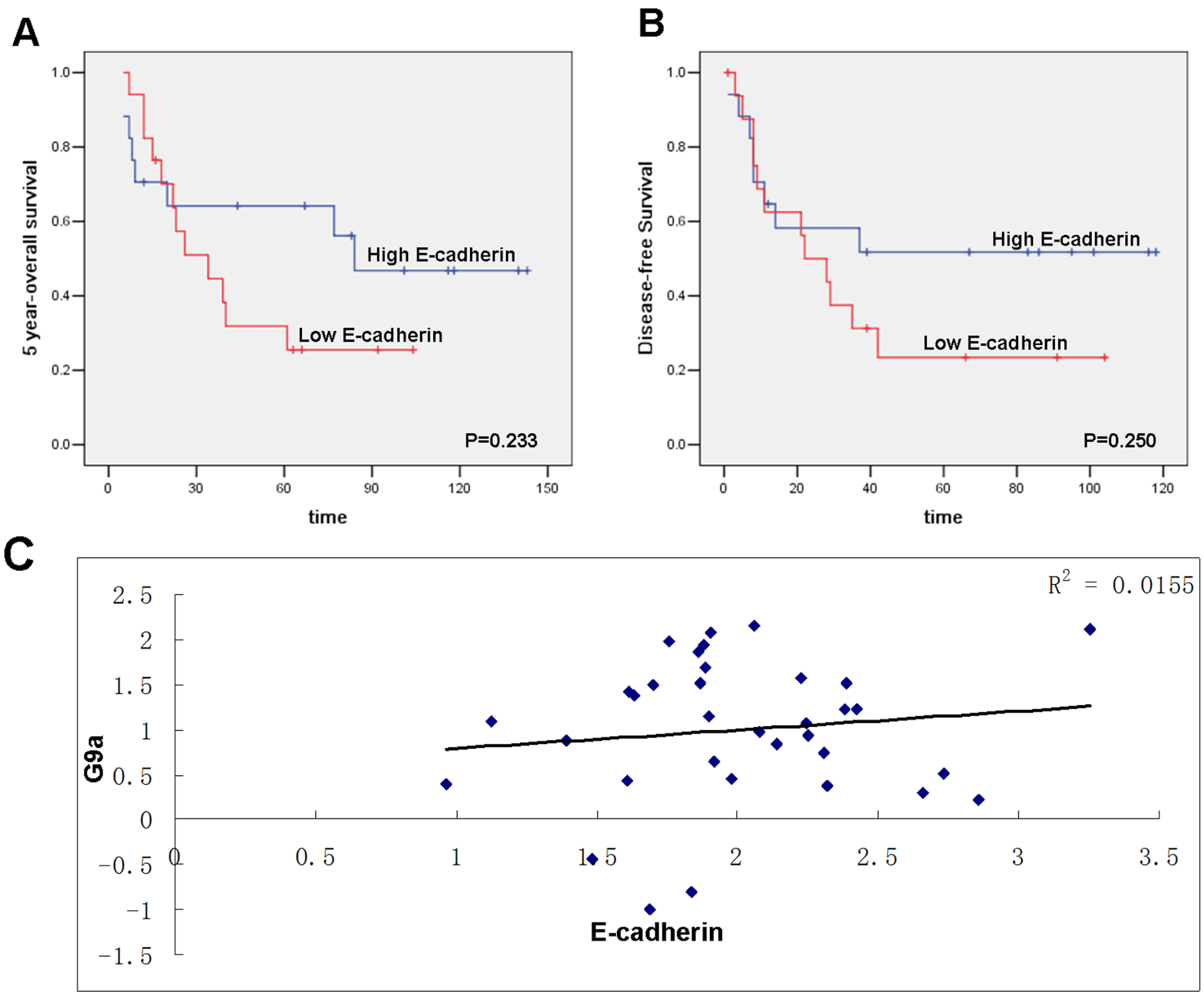

Figure S1: Survival analysis of HNSCC patients depend on E-cadherin expression. (A) Graph demonstrates the 5 year-overall survival in HNSCC patients with low and high E-cadherin expression. **(B)** Graph demonstrates the disease-free survival in HNSCC patients with low and high E-cadherin expression. **(C)** No significant correlation was found between E-cadherin and G9a at the mRNA level in a data set from Oncomine.

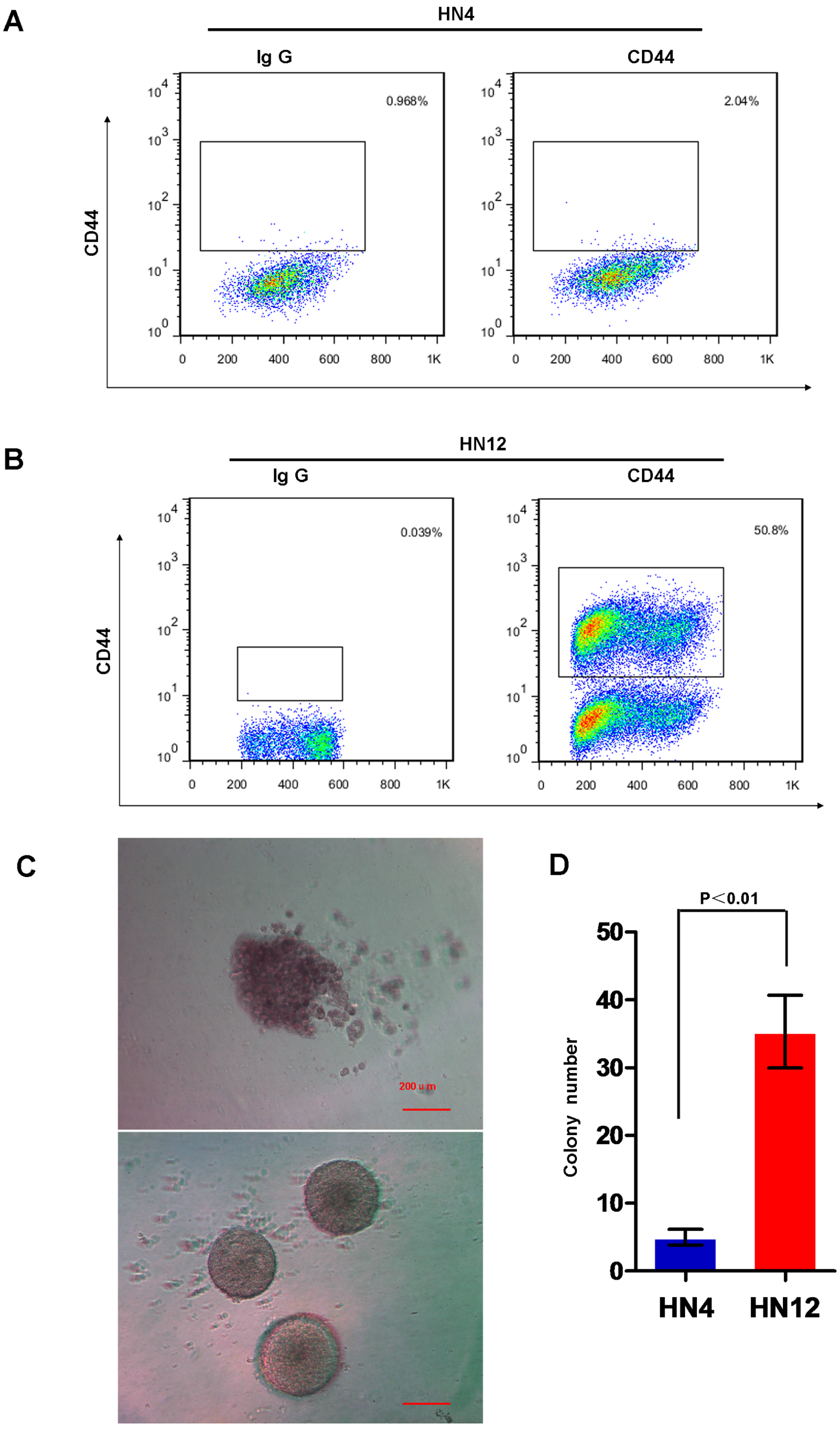

Figure S2: HNSCC metastasic lymph nodes exhibit EMT characters. **(A, B)** FACS analysis of CD44 expression in HN4 and HN12 cells. **(C)** Images demonstrate colonies from anchorage-independent growth (soft agar) analysis of HN4 and HN12 cell. Scale bar = 200 um. **(D)** Graph demonstrates the mean ± SD of colony number in HN4 and HN12 cells from 3 separate experiments.

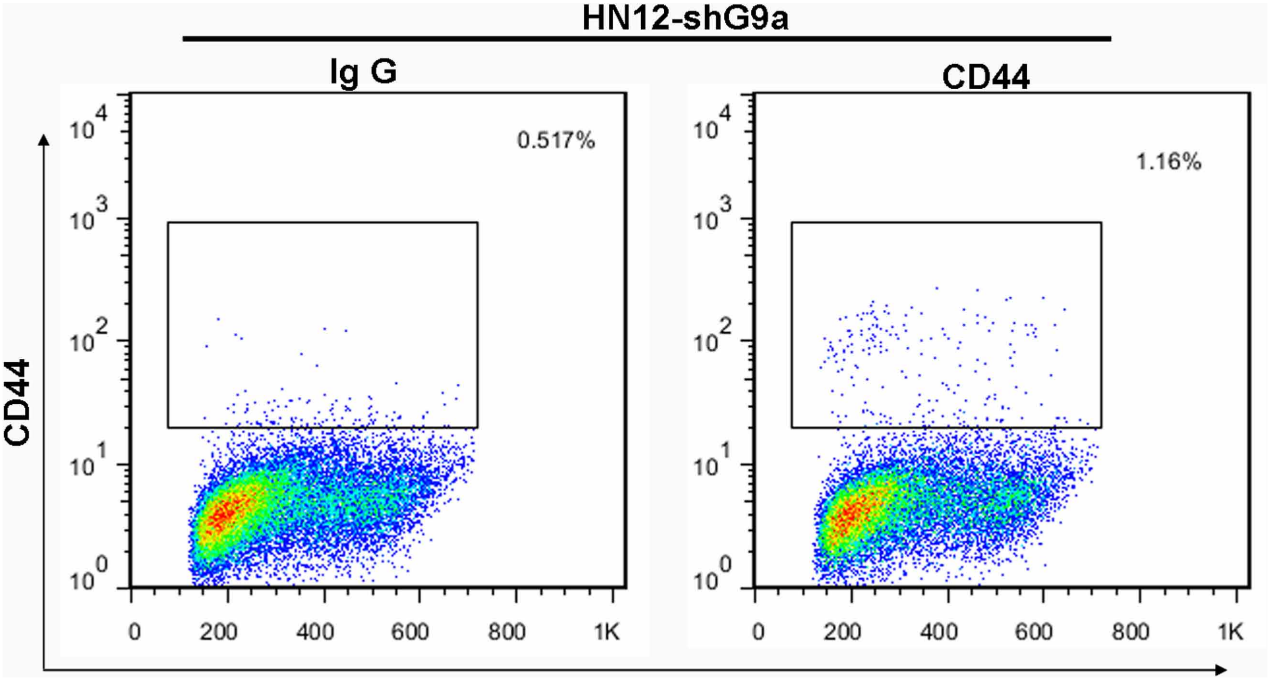

Figure S3: FACS analysis of CD44 expression in HN12 cells stably expressing control vector or G9a shRNA.
